# Draft genome of *Paenibacillus ottowii* LIS04 and *Bacillus velezensis* IM14: dual biocontrol bacterial strains with broad-spectrum antifungal activity

**DOI:** 10.1128/mra.00184-25

**Published:** 2025-05-19

**Authors:** Gisele de Fátima Dias Diniz, Valter Cruz-Magalhães, Sylvia Morais de Sousa Tinoco, Luciano Viana Cota, Ubiraci Gomes de Paula Lana, José Edson Fontes Figueiredo, Lourenço Vitor Silva Ferreira, Vera Lúcia dos Santos, Christiane Abreu de Oliveira-Paiva

**Affiliations:** 1 Department of Microbiology, Federal University of Minas Gerais 28114, Belo Horizonte, Minas Gerais, Brazil; 2 Department of Soil Microbiology, Embrapa Maize and Sorghum 592341 , Sete Lagoas, Minas Gerais, Brazil; 3 Department of Bioengineering, Federal University of São João del-Rei 74383, São João del Rei, Minas Gerais, Brazil; The University of Arizona, Tucson, Arizona, USA

**Keywords:** biological control, plant growth-promoting bacteria, bioproduct, genomics

## Abstract

*Paenibacillus* and *Bacillus* are gram-positive bacteria known for promoting plant growth and controlling agriculturally important phytopathogens. This study highlights the genomic traits of *Paenibacillus ottowii* LIS04 and *Bacillus velezensis* IM14, isolated from sorghum seeds and maize stigmas, respectively, with potential applications in developing innovative agricultural bioproducts.

## ANNOUNCEMENT

*Paenibacillus ottowii* LIS04 and *Bacillus velezensis* IM14 are potent antagonists against several plant pathogens and exhibit plant growth-promoting properties (1–3). These strains produce antifungal secondary metabolites, including iturin, fengycin, and surfactin from *B. velezensis* IMI14 and fusaricidin from *P. ottowii* LIS04 (4). Their compatibility highlights the potential for combined application in developing innovative bioproducts.

*P. ottowii* LIS04 was isolated from sorghum (*Sorghum bicolor* L.) seeds collected in the Cerrado biome (Brazilian savanna) of Sete Lagoas (19.28°S, 44.14°W), Minas Gerais, Brazil. The seeds were surface-disinfected and incubated in germination boxes at 22°C with a 12 hour photoperiod. The strain was obtained from seeds exhibiting bacterial colonies that visibly inhibited the mycelial growth of phytopathogenic fungi. Bacterial samples were collected using sterile platinum loops and streaked onto Tryptone Soy Agar (Kasvi, Brazil) plates. *B. velezensis* IM14 strain was isolated from maize (*Zea mays* L.) stigmas collected in the Cerrado region of Sete Lagoas. The stigmas were macerated and suspended in sterile saline solution (0.9%). Serial dilutions were plated on Potato Dextrose Agar and incubated at 28°C for 48 hours. Both strains were cryopreserved in a glycerol-based medium at −80°C prior to genome sequencing. *Paenibacillus ottowii* LIS04 and *B. velezensis* IM14 were deposited in the Embrapa Collection of Multifunctional and Phytopathogenic Microorganisms for Maize and Sorghum (CMMF) under accession BRM053425 (CMPC2456) and BRM046334 (CMPC2048), respectively.

The DNA extraction of the bacterial isolates was performed using pure cultures grown in Luria Bertani liquid medium at 28°C for 24 hours under agitation at 150 rpm. After this period, the culture was centrifuged at 16,000 rpm, and the supernatant was discarded. Genomic DNA from LIS04 and IM14 was extracted using the Wizard Genomic DNA Purification Kit (Promega, USA) and quantified with a Qubit 2.0 fluorometer (Life Technologies, USA). Whole-genome sequencing (WGS) libraries were prepared according to the BGISEQ-500 WGS protocol described by Huang et al. (5). Genome sequencing was performed on the Illumina HiSeq 4000 platform (Illumina, USA) at the Beijing Genomics Institute (BGI), Shenzhen, China. Sequencing was performed using a 150 bp paired-end strategy. Raw reads were processed with Trimmomatic v0.38 (6) to remove low-quality and adapter sequences (Phred score <20). The quality-filtered reads were then assembled *de novo* using SPAdes v3.12.0 (7). Assembly quality was assessed with QUAST v5.0.2 (8), and genome completeness was evaluated using BUSCO v5.3.1 (Benchmarking Universal Single-Copy Orthologs) (9).

The key genomic features of LIS04 and IM14 are summarized in Table 1. The genomic sequences were submitted to NCBI and annotated using the Prokaryotic Genome Annotation Pipeline v6.6.1 (10). The annotation process identified 4,928 coding sequences, 92 rRNAs, and 109 tRNAs in LIS04, while IM14 contained 3,786 coding sequences, 22 rRNAs, and 84 tRNAs.

**TABLE 1:** Genomic features of *Paenibacillus ottowii* LIS04 and *Bacillus velezensis* IM14

Assembly	*P. ottowii*	*B. velezensis*
Strain ID	LIS04	IM14
Fast ANI placement (%)	96866	98296
Fast ANI reference (NCBI)	GCA_006874425.1	GCA_001461825.1
Library size	6,832,602	6,847,781
Number of contigs	164	55
Total sequence length (pb)	5,553,857	3,997,051
Total ungapped length (bp)	5,553,759	3,996,960
N50 (kb)	473,395	468,182
G+C content (%)	45.5	46.5
Genes	5.209	3.977
Protein-coding	4932	3.794
Noncoding (RNA)	1	2
Genome completeness/contamination (%)	99.7/0.56	99.41/0
Coverage (×)	347	489
SRA identifiers	SRS23177414	SRS23197145
Genome assembly (NCBI)	GCF_030676815.1	GCF_030676595.1

For taxonomic inference based on genomic data, the Type (Strain) Genome Server (https://tygs.dsmz.de) was utilized to identify the *Paenibacillus* and *Bacillus* species most closely related to LIS04 and IM14. The results confirmed that LIS04 belongs to *P. ottowii*, while IM14 corresponds to *B. velezensis*.

## Data Availability

The draft genome sequences of *Paenibacillus ottowii* LIS04 and *Bacillus velezensis* IM14 strains were deposited in GenBank BioProject PRJNA1001033. The Whole Genome Shotgun project has been deposited at DDBJ/ENA/GenBank under the accession nos. NZ_JAUUUV000000000.1 and NZ_JAUUUY000000000.1, with BioSample IDs SAMN36791032 and SAMN36791029, for LIS04 and IM14, respectively. The raw sequencing reads for LIS04 and IM14 have been submitted to the SRA database and are available under accession numbers SRS23177414 and SRS23197145. The versions presented here represent the first releases of the LIS04 and IM14 genomes.

## References

[1] Diniz GFD, Cota LV, Figueiredo JEF, Aguiar FM, da Silva DD, de Paula Lana UG, dos Santos VL, Marriel IE, de Oliveira-Paiva CA. 2021. Antifungal activity of bacterial strains from maize silks against Fusarium verticillioides. Arch Microbiol 204:89. doi:10.1007/s00203-021-02726-4 34962587

[2] Diniz GFD, Figueiredo JEF, Lana UGP, Marins MS, Silva DD, Cota LV, Marriel IE, Oliveira-Paiva CA. 2022. Microorganisms from corn stigma with biocontrol potential of Fusarium verticillioides. Braz J Biol 82:e262567. doi:10.1590/1519-6984.262567 36043660

[3] Diniz GFD, Cota LV, Figueiredo JEF, Lana UGP, Sousa Marins M, Campos Silva F, Santos VL, Oliveira CA. 2023. Isolation and identification of broad-spectrum antagonist bacteria against pathogenic fungi of maize crop. RBMS 22:e1342. doi:10.18512/rbms2023v22e1342

[4] Diniz GFD, Figueiredo JEF, Canuto KM, Cota LV, Souza ASQ, Simeone MLF, Tinoco SMS, Ribeiro PRV, Ferreira LVS, Marins MS, Oliveira-Paiva CA, dos Santos VL. 2024. Chemical and genetic characterization of lipopeptides from Bacillus velezensis and Paenibacillus ottowii with activity against Fusarium verticillioides. Front Microbiol 15:1443327. doi:10.3389/fmicb.2024.1443327 39252841 PMC11381237

[5] Huang J, Liang X, Xuan Y, Geng C, Li Y, Lu H, Qu S, Mei X, Chen H, Yu T, Sun N, Rao J, Wang J, Zhang W, Chen Y, Liao S, Jiang H, Liu X, Yang Z, Mu F, Gao S. 2020. BGISEQ-500 （DNBSEQ-G50）WGS library construction. Protocols.io. doi:10.17504/protocols.io.bim3kc8n

[6] Bolger AM, Lohse M, Usadel B. 2014. Trimmomatic: a flexible trimmer for Illumina sequence data. Bioinformatics 30:2114–2120. doi:10.1093/bioinformatics/btu170 24695404 PMC4103590

[7] Prjibelski A, Antipov D, Meleshko D, Lapidus A, Korobeynikov A. 2020. Using SPAdes de novo assembler. Curr Protoc Bioinformatics 70:e102. doi:10.1002/cpbi.102 32559359

[8] Mikheenko A, Prjibelski A, Saveliev V, Antipov D, Gurevich A. 2018. Versatile genome assembly evaluation with QUAST-LG. Bioinformatics 34:i142–i150. doi:10.1093/bioinformatics/bty266 29949969 PMC6022658

[9] Manni M, Berkeley MR, Seppey M, Zdobnov EM. 2021. BUSCO: assessing genomic data quality and beyond. Curr Protoc 1:e323. doi:10.1002/cpz1.323 34936221

[10] Tatusova T, DiCuccio M, Badretdin A, Chetvernin V, Nawrocki EP, Zaslavsky L, Lomsadze A, Pruitt KD, Borodovsky M, Ostell J. 2016. NCBI prokaryotic genome annotation pipeline. Nucleic Acids Res 44:6614–6624. doi:10.1093/nar/gkw569 27342282 PMC5001611

